# Supplementary material for: Determinants of wheat residue burning: Evidence from India
Source: PLoS One. 2023 Dec 29;18(12):e0296059. doi: 10.1371/journal.pone.0296059 (PMC10756511; doi:10.1371/journal.pone.0296059)
Supplement: S1 Appendix — (DOCX) [file pone.0296059.s001.docx]

**S1 Appendix: Survey Questionnaire^[[1]](#footnote-1)^**

**Socioeconomic Survey for Controlling Crop Residue Burning**

| Date of the interview |  |  |  | Date checked |  |  |  |  |
| --- | --- | --- | --- | --- | --- | --- | --- | --- |
| Signature of enumerator: |  | | | Signature of supervisor: |  | | |  |
| Time started: |  | | | Time ended: |  | | |  |
| Village name: |  | | | Household ID#: |  | | |  |
| Household GPS/landmark |  | | | | | | |  |

1. **Household Composition and Farm Characteristics**

| 1.1 | Father’s name |  | | | | | |
| --- | --- | --- | --- | --- | --- | --- | --- |
| 1.2 | Respondent Mobile no. |  | | | | | |
| 1.3 | Relationship of respondent with household head |  | | | | | |
|  | | 1.4.1 Name | 1.4.2 Gender  Code A | 1.4.3 Age  Years | 1.4.4 Education Code B | 1.4.5 Primary job Code C | 1.4.6 Secondary job Code C |
| 1.4 Respondent (*person making farming decision*) | |  |  |  |  |  |  |

| 1.5 | Type of house construction/material | (Code: 1- pukka, 2- semi-pukka, 3 - kuccha) | |
| --- | --- | --- | --- |
|  | Type of harvesting and sowing machinery | *Owned (Y/N) – (1/0)* | *Available for hire (Y/N) – (1/0)* |
| 1.6 | Tractor |  |  |
| 1.7 | Seeder |  |  |
| 1.8 | Thrasher |  |  |
| 1.9 | Driller |  |  |
| 1.10 | Harvester |  |  |
|  | Number of livestock units owned | 1.11 Buffaloes |  |
|  |  | 1.12 Cows |  |
|  |  | 1.13 Bullocks |  |
|  |  | 1.14 Goats |  |
|  |  | 1.15 Others |  |
|  | Land units | *Cultivated* | *Owned* |
| 1.16 | Land units for paddy in 2018 |  |  |
| 1.17 | Land units for **soft** wheat in 2018 – 2019 |  |  |
| 1.18 | Land units for **coarse** wheat in 2018 – 2019 |  |  |
| 1.19 | Land units for other crops in 2018 - 2019 |  |  |
| **Code A:** 1 – Male; 2 – Female  **Code B**: 0 – No education/illiterate; 1 – semi-literate (never attended school); 2 – Primary (Grade 1-5); 3– Middle (Grade 6-8); 4 – Secondary (Grade 9-10); 5 – Higher Secondary (Grade 11-12); 6 – Graduate; 7 – Post graduate; 8 – Vocational education  **Code C :**1– Farming; 2 – Livestock rearing; 3 – Salaried employment; 4 – Self-employed off farm; 5 – Casual labourer on farm; 6 – Casual labourer off farm; 7 – Involved in household chores; 99 – Other (specify) | | | |

1. **Crop Residue Management**

| 2.1 | Is burning paddy residue a common practice in your village? | - Not at all - Occasionally - Often - Very common - Everybody does it | 1  2  3  4  5 |
| --- | --- | --- | --- |
| 2.2 | Is burning wheat residue a common practice in your village? | - Not at all - Occasionally - Often - Very common - Everybody does it | 1  2  3  4  5 |
| 2.3 | What was the time period of wheat residue removal in your village in April/May 2018? | Start date |  |
|  |  | End date |  |
| 2.4 | In the last 5 years, was wheat residue ever burned on your land? | Yes/ No/ Unwilling to Answer  (1/ 0 /99) | |
| 2.5 | If yes to 2.4 *--* How many times was wheat residue burned on your land? | *Specify how many times in last 5 years*: |  |
| 2.6 | Did you burn wheat residue this season? | Yes/ No/ Unwilling to Answer  (1/ 0 /99) | |
| 2.7 | If yes to 2.6 specify how many acres? |  | |
| 2.8 | If yes to 2.6, why do you choose to burn?  If no to 2.6, what do you think is the most important factor why farmers choose to burn? | No other use | 1 |
|  |  | Expensive to hire machine | 2 |
|  |  | Expensive to hire labor | 3 |
|  |  | Combine harvester | 4 |
|  |  | Improve soil quality | 5 |
|  |  | Other (specify): | 6 |
| 2.9 | When you do not burn wheat residue, which method do you use to remove it? | *Labor Removal (1)* | Mixed of both methods (3)  Use labor for ____% of total residue. |
|  |  | *Machine Removal (2)* |  |
| 2.10 | When you do not burn wheat residue, what do you do with the residue? | Reincorporate into soil | 1 |
|  |  | Leave it on the side of the farm | 2 |
|  |  | Sell the residue | 3 |
|  |  | Fodder for cattle | 4 |
|  |  | Cooking fuel | 5 |
|  |  | Other (specify): | 6 |
|  | Please estimate the time and cost of removing wheat residue **per acre** of land | 2.11 Total cost of machine removal **per acre** |  |
|  |  | 2.12 Total cost of labor removal **per acre** |  |
| 2.13 | How many quintals of wheat residue are generated per acre? (Leave blank if don’t know) |  | |
| 2.14 | What is the selling price of wheat residue per quintals? (Leave blank if don’t know) |  | |
| 2.15 | How do you think burning wheat residue affects soil quality? (tick response) | Improves soil quality | 1 |
|  |  | Diminishes soil quality | 2 |
|  |  | No effect / don’t know | 3 |
| 2.16 | Are you aware of negative environmental effects of wheat residue burning? | Y/N (1/0) |  |
| 2.17 | Are there any programs to reduce wheat residue burning that you are participating in? | Y/N (1/0) |  |
| 2.18 | If Yes to 2.17, then what type of program is it? | Awareness | 1 |
|  |  | Machine availability | 2 |
|  |  | Cash subsidy | 3 |

1. The translated Hindi version used by the enumerators is available upon request. [↑](#footnote-ref-1)
