# Supplementary material for: Determinants of wheat residue burning: Evidence from India
Source: PLoS One. 2023 Dec 29;18(12):e0296059. doi: 10.1371/journal.pone.0296059 (PMC10756511; doi:10.1371/journal.pone.0296059)
Supplement: Attachment — Submitted filename: Reviewer Comments and Responses.docx [file pone.0296059.s003.docx]

**Reviewer Comments and Author Responses**

The authors would like to thank the Editor and the three Reviewers for their time spent reviewing the manuscript and for the chance to revise the manuscript by incorporating their suggested changes to the narrative of the Introduction, Methodology, Discussion, and Conclusion sections. Their constructive comments have helped to improve the manuscript to its present form. The detailed explanations of these major revisions are provided in the responses to each of the Reviewers’ comments that follow.

- Reviewer 1’s comments and detailed author’s responses are listed on pages 2-4 in this document.
- Reviewer 2’s comments and detailed author’s responses are listed on pages 5-7 in this document.
- Reviewer 3’s comments and detailed author’s responses are listed on pages 8-9 in this document.

**Review Comments to the Author**

Please use the space provided to explain your answers to the questions above. You may also include additional comments for the author, including concerns about dual publication, research ethics, or publication ethics. (Please upload your review as an attachment if it exceeds 20,000 characters)

**Reviewer #1**:

**Manuscript ID: PONE-D-23-10161 Journal Name: PLOS ONE Manuscript title: Determinants of Wheat Residue Burning: Evidence from India. The manuscript is based on survey data collected over two years from India and analysis of the surveyed data. The work has been done on a noble topic which is less documented in the literature. The findings could be useful for managing the crop residue burning of wheat that could be a major problem in future. The manuscript is well-written and I have enjoyed reading the article. However, a few things need to be amended before publication.**

**1. The introduction seems to be lengthy and needs to be shortened. A few paragraphs more resemble “results and discussion” and need to be placed appropriately.**

**2. The data collection has been done by a service provider and authors are not directly involved in the data collection process. Hence, I am eager to know the validity of the collected data or the authenticity of the data. Was it validated by the authors, if yes how?**

**3. A paragraph describing the limitations of the study needs to be added.**

**4. The conclusion seems very lengthy and contains many references. Conclusions should be concise and based on the understanding of the authors on results and discussions. It now looks like the discussion with cited references.**

**5. The article needs to be checked thoroughly for writing, punctuation marks, etc. The article may be accepted after “Major revision”.**

**Authors’ response:**

We thank Reviewer 1 for their insightful and constructive comments. As suggested, the Introduction section has been shortened and the problem statement is made clearer. It is renamed as Introduction and Motivation. Background on CRB in India has been placed as a separate section from the Introduction. Furthermore, the paragraph that previously summarized the results of the paper in the Introduction has now been placed in the Results section of the revised manuscript.

We outline the following aspects concerning the point raised about the validity of the collected data or its authenticity. The data collection agency contracted by this project’s grant money was the same agency as used previously by the same authors of the previous study on rice residue burning by a larger sample of farmers in the same villages and district of Haryana. The agency’s standard operating procedure involves rechecking of data entry of the survey by supervisors of data collectors on the field.

- We checked for wheat and rice burning pattern in the data and look for any anomalies.
- There is a paper record of each survey response to check for authenticity.
- Two of the authors visited the field site and interacted with the agency and trained its enumerators.
- The data are quality checked at each stage of data collection. For example – if certain data seemed incorrect or anomalous, the respondents were contacted again by phone.

Moreover, the said data collection agency has extensive field experience in primary data collection at the village household level across several Indian states and in this geography.

We thank the reviewer for the suggestion to discuss the limitations of the study. We have amended the last paragraph of the Conclusion section to point out the limitations of our study (Page 25).

As suggested by the Reviewers, the cited references have been removed from the Conclusion section and these have been placed in a new Discussion section prior to it. The Conclusion section focuses on the study’s inferences.

We have checked the revised manuscript for writing and punctuation marks, and also done a spellcheck for any errors.

**Reviewer #2:**

**The introduction and background section of the paper need to be refined further to add more clarity in the 'statement of problem'. Also, include a few more studies especially on residue management/CRB practices of rice in the western as well as southern regions of India. Explain the research design and sampling pattern briefly in the methodology. Results of the study are promising though the sample size is small. Results and discussion of 3.2 could be highlighted more with some new insights from the study in place of what is known already. Conclusion needs a major revision. Be more precise in presenting the inferences of the study rather than giving a summary of the work in conclusion. Try not to include any references in this session.**

**Authors’ response:**

We thank Reviewer 2 for their insightful and constructive comments. As suggested, the Introduction section has been shortened and the problem statement is made more succinct. It is renamed as Introduction and Motivation. Background on CRB in India has been placed as a separate section following the introduction. Furthermore, the paragraph summarizing the results of the paper has been placed in the Results section.

With regards to crop residue burning taking place in other parts of India, we have added more references on crop residue burning occurrences in western and southern India. See Footnote 1 on Page 2 - “Similarly, attention has not focused on other parts of India where crops such as rice, sugarcane, and oilseed are also subject to postharvest residue burning; for example, in western and southern India in states such as Maharashtra, Karnataka, and Tamil Nadu [16, 21, 23].”

Regarding the research design and sampling, we have expanded upon these in the revised manuscript in the Data section and the Methodology section. In the Data section, we have added more details on the sampling for our study. The following sentences have been added to Pages 6-7 of the revised manuscript:

“Our target was 40 farmers per village. During the selection process, we excluded farmers who only grew the non-coarse varieties of rice (i.e., basmati) or non-paddy crops since the residues of these crops typically are not burnt. This process resulted in between 35 to 40 farmers for each village for a total of 301 farmers, who grew coarse varieties of rice among other things, participating in the 2019 survey.

The random sampling for this research was designed to answer the questions of whether choosing CRB made economic sense for wheat farmers, and if there is a relationship in the residue management choices between rice and wheat.”

The Methodology section is divided into two parts – Benefit-cost analysis framework and Regression framework. These sections describe the analytical framework that is used to investigate the causes of wheat residue burning.

Regarding the size of the sample for this study, we have added an acknowledgement of this aspect as a limitation. Budgetary limitations permitted a sample size of 301 farmers to be interviewed. We have added a paragraph on Page 25 in the Conclusion section of the revised manuscript, wherein we state that we cautiously extrapolate and generalize the findings from this study to the wider context of wheat crop residue burning in India.

With regards to highlighting with new insights from the study in place of what is known already, the revised manuscript has a new Discussion section following the Results section on Pages 22-24. The Discussion section highlights the insights and policy implications from our study.

As suggested by the Reviewers, the references have been removed from the Conclusion section, and these have been placed in the Discussion section prior to it. The Conclusion section focuses more on the study’s inferences.

**Reviewer #3**:

**The data in the manuscript support the conclusions drawn. The experiment seems rigorous enough; whereas, the results of perceived effect of residue burning on soil quality and awareness of farmers on negative environmental effects seem to have not been captured properly.**

**Authors’ response:**

We thank Reviewer 3 for their insightful and constructive comments. As pointed out by the reviewer, the perceived effect of residue burning on soil quality and awareness of the negative environmental effect of crop residue burning (CRB) are two of the relevant factors expected to influence the residue burning decision of the farmers. The negative effect of burning on soil quality imposes a private cost on the farmer, whereas the negative effect on the environment is more of an external social cost. In our previous study, which analyzes the factors influencing paddy residue management practices (reference [17]), we find that farmers who perceive residue burning as diminishing soil quality are statistically significantly less likely to do so; however, awareness of negative environmental effects of CRB does not influence the choice to burn. In our current study, we include the perceived effect of CRB on soil quality and the environment at each level of our analysis.

In Table 2, we compare socioeconomics and other aspects among these two groups of farmers – those who undertook wheat CRB (Yes) and those who did not (No). Table 2 shows that the two groups of farmers significantly differ in their perceptions relating to the effects of residue burning on soil quality and the environment based on Fisher’s Exact test of the equality of proportions. A greater proportion of farmers who did not burn wheat were aware of CRB’s negative implications for soil health and environmental outcomes. These relationships are visually presented in Figure 1. Both variables are included in each of our five regression models. In the regression analysis, we have tried to control for an extensive list of relevant demographics, socio-economic, and perception-based variables.

As we discuss in the Methodology section and Results section, after controlling for different potential determinants of CRB, we find that “the perception of the effects of CRB on soil quality has a significant impact on farmer behavior. Farmers who think that CRB diminishes soil quality are significantly less likely to choose CRB than those who think it improves soil quality or are uncertain about its effect. This result suggests that farmers do internalize their associated costs and benefits of CRB. Farmer’s awareness of the negative environmental impact of CRB has a negative sign as one would expect; the effect however is statistically insignificant. Thus, farmers do not substantially internalize the external social costs of CRB in their private decisions”. These findings are consistent with our previous findings concerning rice CRB. We have thus tried to best account for the farmers’ perceived effects of residue burning on soil quality and the awareness of farmers on the negative environmental effects in our regression framework.
